# ACLA-Net: attention-guided cross-level alignment network for MRI-based prostate cancer lesion segmentation

**DOI:** 10.3389/fphys.2026.1860527

**Published:** 2026-09-07

**Authors:** Bin Cai, Yunfu Zeng, Jiang Liu, Zhu Jiang, Hong Fan

**Affiliations:** 1 Department of Radiology, Yibin First People’s Hospital, Yibin, China; 2 School of Mechanical Engineering, Sichuan Polytechnic University, Deyang, China

**Keywords:** cross-level feature alignment module, prostate cancer lesion segmentation, SE-guided context aggregation module, SE-guided multi-kernel depthwise module, spatial attention-guided fusion module

## Abstract

**Introduction:**

Benefiting from its radiation-free property and excellent soft-tissue contrast, magnetic resonance imaging (MRI) has become a crucial modality for prostate cancer diagnosis. However, automatic segmentation of prostate cancer lesions in MRI is still a challenging task due to the considerable variations in lesion morphology and scale, as well as the often indistinct boundaries between tumors and surrounding tissues. To address these challenges, we propose ACLA-Net, an attention-guided cross-level alignment network tailored for MRI-based prostate cancer lesion segmentation.

**Methods:**

Specifically, ACLA-Net is built upon the U-Net architecture. To enhance the interaction and alignment of encoder features across different semantic levels, a cross-level feature alignment module (CLFAM) is introduced into the second, third, and fourth skip connections. Meanwhile, a spatial attention-guided fusion module (SAFM) is incorporated into the first skip connection to emphasize discriminative spatial cues and improve the fusion of shallow features. At the bottleneck stage, a SE-guided context aggregation module (SECAM) is employed to capture richer global contextual information and strengthen high-level semantic representation. Furthermore, the conventional double convolution blocks in the encoder are replaced with the SE-guided multi-kernel depthwise module (SEMKDM), which improves multi-scale feature extraction while reducing computational redundancy.

**Results and discussion:**

To evaluate the effectiveness and generalization ability of the proposed method, extensive experiments were conducted on two self-constructed prostate cancer MRI datasets, namely ProstateCancer-T2WI and ProstateCancer-ADC, as well as the public DDTI thyroid dataset. On ProstateCancer-T2WI, ACLA-Net achieved Dice, MCC, and Jaccard scores of 0.7798, 0.7782, and 0.6403, while on ProstateCancer-ADC, it obtained corresponding scores of 0.8332, 0.8328, and 0.7159. Furthermore, on the public DDTI dataset, ACLA-Net attained Dice, MCC, and Jaccard scores of 0.7752, 0.7438, and 0.6338. In addition, ablation studies were carried out to verify the effectiveness of each proposed module. The experimental results demonstrate that ACLA-Net achieves reliable segmentation performance on prostate cancer MRI and favorable generalization on the public dataset, suggesting its potential to provide useful support for lesion delineation and subsequent clinical assessment.

## 1 Introduction

Prostate cancer is one of the most common malignant tumors in the male genitourinary system and poses a serious threat to the health and survival of middle-aged and elderly men. In recent years, with the intensification of population aging and the continuous advancement of screening techniques, both the incidence and detection rate of prostate cancer have shown an increasing trend. This disease is characterized by marked heterogeneity: some cases progress slowly, whereas others exhibit strong aggressiveness. Such aggressive tumors may not only damage the prostate and its surrounding normal tissues, but may also lead to local invasion and even distant metastasis, thereby substantially affecting patient prognosis and quality of life. The clinical manifestations of prostate cancer are relatively complex. In the early stage, obvious symptoms are often absent. However, as the disease progresses, patients may gradually develop urinary frequency, urinary urgency, dysuria, hematuria, and bone pain. In severe cases, the disease can further impair physical function and overall health status. At present, the clinical management of prostate cancer generally relies on a multidisciplinary treatment strategy, including surgical resection, radiotherapy, endocrine therapy, and chemotherapy, with the aims of controlling tumor progression, alleviating symptoms, and prolonging survival. Therefore, early screening, accurate diagnosis, and timely therapeutic intervention are of great importance for improving prognosis and enhancing the quality of life of patients with prostate cancer.

At present, magnetic resonance imaging (MRI) has become an indispensable imaging modality for the diagnosis and clinical management of prostate cancer. Research and clinical evidence indicates that MRI not only provides reliable support for lesion detection and localization, but also plays an important role in targeted biopsy, local staging, active surveillance, treatment planning, and post-treatment recurrence assessment. Therefore, accurate segmentation of the prostate and cancerous lesions is of great significance for improving diagnostic consistency and enhancing the reliability of subsequent quantitative analysis. Traditionally, prostate cancer MRI segmentation has mainly relied on manual delineation. However, this process is not only labor-intensive and time-consuming, but also highly dependent on the experience and subjective judgment of radiologists, leading to considerable inter-observer variability. In the early stage of automatic segmentation research, traditional image processing methods were widely adopted to extract the prostate and lesion regions, including region growing (Mazonakis et al., 2001), multi-atlas-based (Acosta et al., 2017), and Markov random field (Liu et al., 2009). Although these methods can achieve acceptable performance when the target structures are relatively regular and the boundaries are clear, they often fail to provide stable and accurate segmentation in prostate MRI due to challenges such as intensity inhomogeneity, indistinct lesion boundaries, substantial morphological variation, and strong interference from surrounding tissues. Moreover, conventional methods usually rely on handcrafted features, initialization strategies, or prior assumptions, which limits their robustness and generalization ability in complex scenarios.

In recent years, with the rapid advancement of deep learning, convolutional neural network-based approaches have demonstrated remarkable potential in prostate cancer MRI segmentation. Compared with conventional image processing techniques, deep learning models are capable of automatically learning hierarchical feature representations in an end-to-end manner, thereby reducing reliance on handcrafted features and complex prior knowledge while enabling more robust and accurate target delineation in challenging imaging scenarios. Among these methods, encoder-decoder architectures represented by fully convolutional networks and U-Net variants have been widely adopted for prostate and prostate cancer lesion segmentation owing to their strong capability in feature extraction and multi-scale information integration. For example, Zhang et al. (2019) proposed a dual-attention adversarial network, which jointly models global contextual information and local detailed features through a dual-attention mechanism. Chahal et al. (2022) proposed a segmentation framework built upon U-Net and Xception, where the powerful encoder-decoder structure of U-Net was integrated with the efficient feature learning capability of Xception to strengthen multi-level feature representation and achieve better segmentation results. Liu et al. (2021) introduced squeeze-and-excitation (SE) layers into both the encoder and decoder, and enhanced the network’s ability to respond to important information by adaptively recalibrating channel-wise features. Zhang et al. (2022) developed a cross-modal self-attention distillation module, which enables the transfer of more salient and discriminative features while preserving richer detailed information. Li et al. (2023) developed a connection mechanism within a generative adversarial network and incorporated a contrastive learning module to improve boundary awareness, enabling the model to generate finer and more precise edge representations. Chen et al. (2025) introduced an improved content-guided attention feature fusion module to combine deep semantic information with shallow spatial details, which strengthened multi-level feature interaction and contributed to more accurate segmentation results.

Although existing deep learning methods have achieved notable progress in prostate cancer MRI segmentation, they still face several challenges. On the one hand, prostate cancer lesions are often small, highly variable in shape, and heterogeneous across patients, making it difficult for models to stably capture discriminative lesion features. On the other hand, the intensity differences between tumors and surrounding normal tissues in MRI are often subtle, while ambiguous boundaries, insufficient contrast, and noise interference are also prominent, which easily lead to missed detections, false positives, or inaccurate boundary localization in segmentation results. Moreover, although U-Net-based methods can fuse multi-level features to some extent, their skip connections typically use direct concatenation, which lacks effective cross-level alignment and selective fusion. Meanwhile, conventional encoder convolutions still have limitations in modeling complex multi-scale contextual information, particularly in balancing local detail preservation and global semantic representation. To address these limitations, many recent studies have incorporated various attention mechanisms (Li et al., 2026; Yang et al., 2025; Wang et al., 2025) and context aggregation strategies (Jin et al., 2025; Ji et al., 2024; Yue et al., 2024) into deep learning frameworks, which has led to encouraging improvements in segmentation performance.

Motivated by the above observations, we propose ACLA-Net, an attention-guided cross-level alignment network for MRI-based prostate cancer lesion segmentation. Extensive experiments on two self-constructed prostate cancer MRI datasets demonstrate the effectiveness of our method. The main contributions of this work are summarized as follows:

1. We propose a novel prostate cancer lesion network, which is built upon the U-Net architecture and is specifically designed to enhance cross-level feature interaction, strengthen discriminative spatial representation, and improve multi-scale contextual modeling.
2. A cross-level feature alignment module is introduced into the second, third, and fourth skip connections to enhance the interaction, alignment, and fusion of encoder features across different semantic levels, thereby improving the utilization of complementary multi-scale information.
3. A spatial attention-guided fusion module and a SE-guided context aggregation module are incorporated into the skip connection and bottleneck stage, enabling the network to emphasize discriminative spatial cues while capturing richer global contextual dependencies.
4. The conventional double convolution blocks in the encoder are replaced with the SE-guided multi-kernel depthwise module, which strengthens multi-scale feature extraction with lower computational redundancy.

## 2 Methods

### 2.1 Network architecture of ACLA-Net

**Figure 1:**
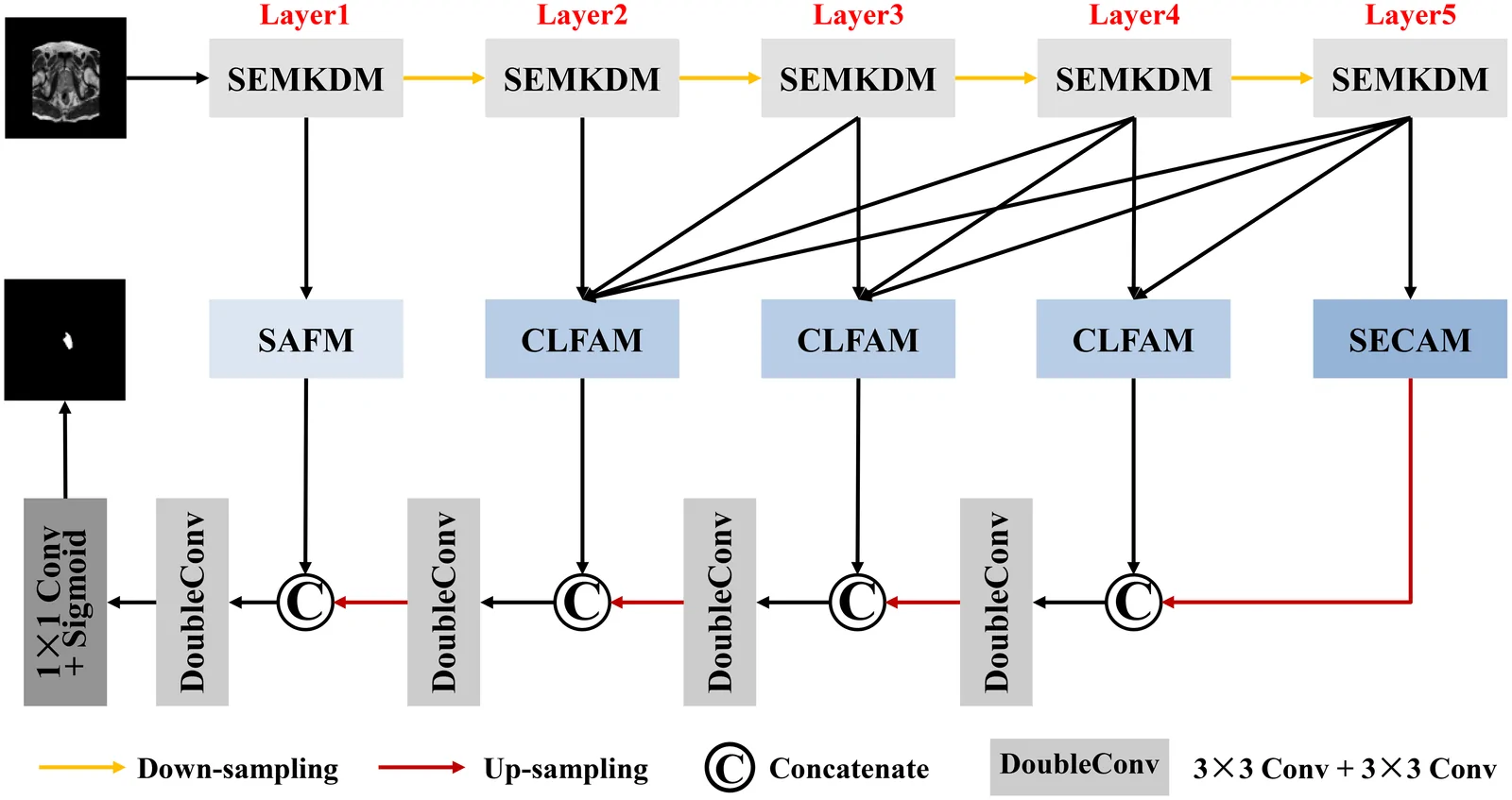
Network architecture of ACLA-Net.

Although the conventional U-Net (Ronneberger et al., 2015) has achieved remarkable success in medical image segmentation, it still has several limitations when applied to MRI-based prostate cancer lesion segmentation. On the one hand, the standard convolutional blocks in the encoder are insufficient for capturing complex multi-scale contextual information from lesions with large variations in size, shape, and boundary appearance. On the other hand, the skip connections in U-Net usually adopt direct concatenation between encoder and decoder features, which ignores the semantic discrepancy across different feature levels and limits the effective utilization of complementary information. To address these issues, we propose ACLA-Net, an attention-guided cross-level alignment network for MRI-based prostate cancer lesion segmentation, as shown in **Figure 1**. Specifically, ACLA-Net follows an encoder-decoder architecture based on U-Net. In the encoder, the conventional convolutional blocks are replaced by the proposed SE-guided multi-kernel depthwise module. By integrating multi-kernel depthwise convolutions with SE-based channel recalibration, SEMKDM enhances the extraction of discriminative multi-scale features while reducing computational redundancy. Through progressive down-sampling, the encoder is able to generate hierarchical feature representations with increasingly rich semantic information. To improve the effectiveness of feature transmission between the encoder and decoder, different skip connection strategies are employed at different stages. In the first skip connection, a spatial attention-guided fusion module is introduced to refine shallow encoder features before they are delivered to the decoder. Since shallow features contain abundant spatial and boundary information, SAFM helps the network focus on informative regions and suppress irrelevant background responses, thereby improving the fusion quality of low-level details. In the second, third, and fourth skip connections, a cross-level feature alignment module is incorporated. Unlike conventional one-to-one skip fusion, CLFAM aggregates and aligns encoder features from different levels, enabling more effective interaction among multi-scale representations. In this way, the semantic gap between shallow and deep features can be alleviated, and the complementary information across different encoder stages can be better exploited. At the bottleneck stage, a SE-guided context aggregation module is embedded to further strengthen contextual modeling. SECAM aggregates global contextual dependencies and enhances high-level semantic features, which is particularly beneficial for the segmentation of prostate cancer lesions with ambiguous boundaries and complex appearance variations. In the decoder, the up-sampled features are concatenated with the corresponding refined skip features and then processed by convolutional blocks to progressively recover the spatial resolution. Finally, a 1×1 convolution followed by a sigmoid activation is used to generate the final lesion segmentation map. Unlike conventional attention-based U-Net variants that mainly refine features within individual stages, ACLA-Net integrates SEMKDM, SAFM, CLFAM, and SECAM within a unified encoder-decoder framework to promote cross-level interaction and stage-specific feature enhancement. This design enables more effective exploitation of multi-scale, spatial, semantic, and contextual information, thereby improving the representation of prostate cancer lesions with complex morphology and indistinct boundaries.

### 2.2 SE-guided multi-kernel depthwise module

**Figure 2:**
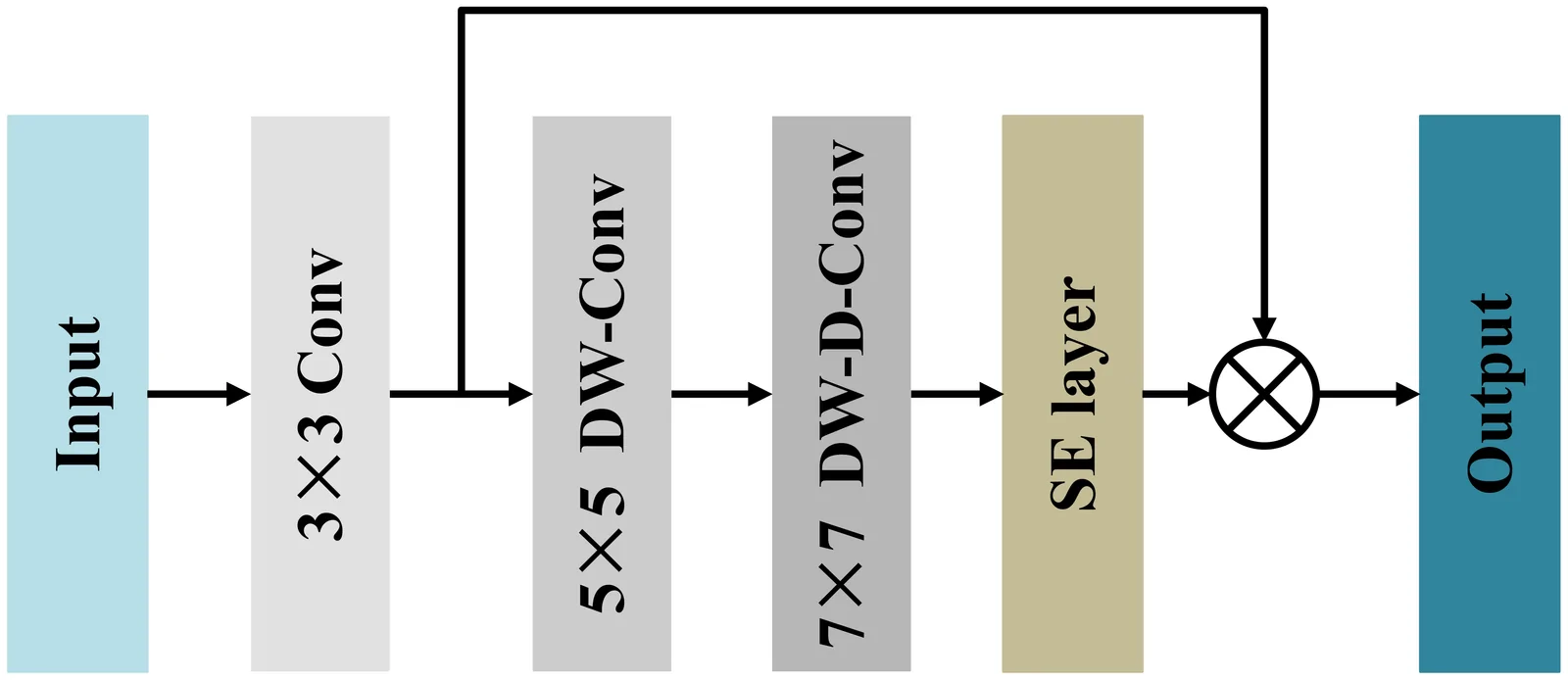
Structure of SE-guided multi-kernel depthwise module.

The conventional convolutional block in U-Net is effective for basic feature extraction, but it still has limitations in capturing diverse contextual information from prostate cancer lesions in MRI. In particular, prostate cancer lesions usually exhibit substantial variations in size, shape, and boundary appearance. Under such circumstances, a single standard convolution is often insufficient to simultaneously model local details and broader contextual dependencies. To address this issue, we design a SE-guided multi-kernel depthwise module, as shown in **Figure 2**, to enhance the multi-scale representation capability of the encoder while maintaining computational efficiency. Specifically, given an input feature map X, SEMKDM first applies a 3×3 convolution to extract preliminary local features. The resulting feature map is then fed into a 5×5 depth-wise convolution (DW-Conv) and a 7×7 depth-wise dilated convolution (DW-D-Conv) in sequence. The use of different kernel sizes and dilation-based receptive field expansion enables the module to capture richer multi-scale contextual information and better adapt to lesion regions with complex morphology. After multi-scale feature extraction, a squeeze-and-excitation (SE) layer is introduced to adaptively recalibrate channel-wise responses. By explicitly modeling inter-channel dependencies, the SE layer enhances informative feature channels and suppresses less useful ones, thereby improving the discriminative representation of lesion-related features. Meanwhile, a shortcut branch is preserved from the output of the initial 3×3 convolution. Finally, the recalibrated feature map is combined with the shortcut feature through element-wise multiplication to generate the final output. Compared with the conventional double-convolution block, SEMKDM combines multi-kernel depth-wise convolutions with SE-based channel recalibration to capture broader multi-scale contextual information while emphasizing informative feature channels. The whole process of SEMKDM is defined in Equation (1):

(1) $$\begin{array}{l} F_{s}=Conv_{3\times 3}(X)\\ Y=F_{s\;}⊗SE(DW-D-Conv_{7\times 7}(DW-Conv_{5\times 5}(F_{s}))) \end{array}$$

where ⊗denotes element-wise multiplication, and Y represents the output feature map.

**Figure 3:**
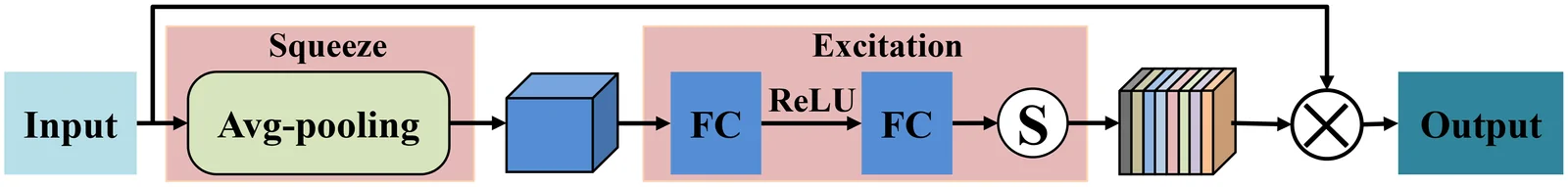
Structure of SE layer.

As illustrated in **Figure 3**, the SE layer (Xiong et al., 2024; Jiang et al., 2022) is introduced to enhance channel-wise feature representation in an adaptive manner. Specifically, average pooling is first used to compress spatial information into channel descriptors, which are then passed through two fully connected layers with nonlinear activations to generate channel-wise attention weights. Finally, these weights are applied to the input feature map to emphasize informative channels and suppress less useful ones. In this way, the SE layer helps improve the discriminative representation capability of the extracted features.

### 2.3 Spatial attention-guided fusion module

**Figure 4:**
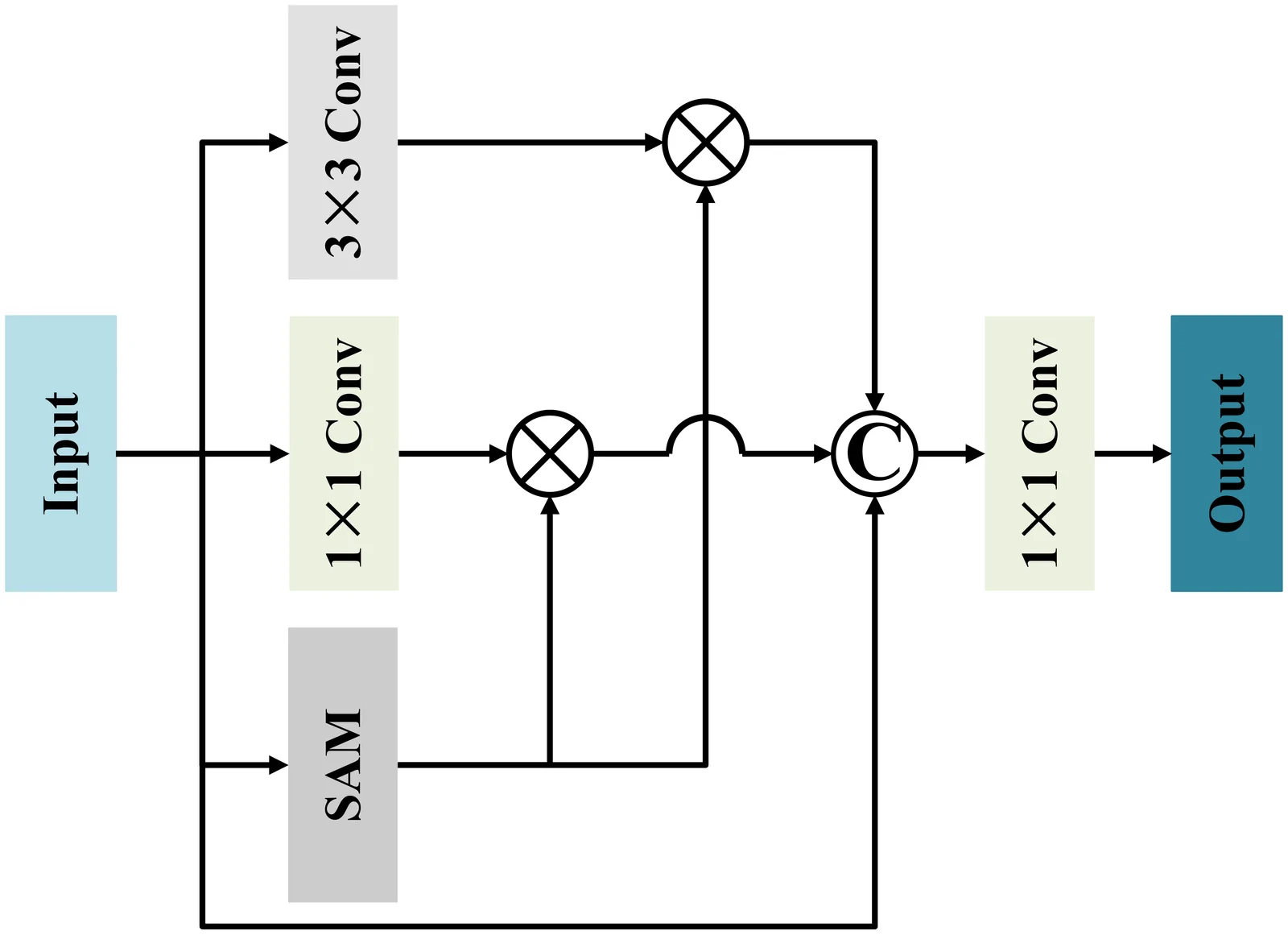
Structure of spatial attention-guided fusion module.

In prostate cancer MRI segmentation, shallow encoder features usually contain rich spatial details and boundary information, which are important for accurate lesion localization. However, these low-level features are also easily affected by background noise and irrelevant responses. If they are directly transmitted to the decoder, the fused features may contain redundant information, which can negatively influence the final segmentation performance. To address this issue, a spatial attention-guided fusion module is introduced into the first skip connection, as shown in **Figure 4**. Specifically, given an input feature map X, SAFM first generates three feature branches. The first branch applies a 3×3 convolution to extract local spatial features. The second branch uses a 1×1 convolution to obtain a compact feature representation and enhance channel interaction. The third branch employs a spatial attention module (SAM) to produce a spatial attention map, which is used to guide the feature refinement process. The output of the 1×1 convolution branch is first modulated by the spatial attention map through element-wise multiplication, enabling the network to emphasize informative spatial responses and suppress irrelevant background interference. Meanwhile, the output of the 3×3 convolution branch is also reweighted by the same attention map, further enhancing discriminative local details. After attention guidance, the refined features from the two convolution branches are concatenated together with the original input feature preserved through a shortcut branch. In this way, SAFM not only highlights lesion-related spatial cues, but also retains the original low-level structural information. Finally, a 1×1 convolution is applied to fuse the concatenated features and generate the module output. Unlike conventional skip connections that directly transfer shallow encoder features to the decoder, SAFM employs spatial attention-guided multi-branch refinement before feature fusion. This design selectively enhances lesion-related spatial responses while suppressing irrelevant background information, thereby preserving more informative boundary and structural cues for subsequent decoding. The overall process of SAFM is defined in Equation (2):

(2) $$\begin{array}{l} A=SAM(X)\\ Y=Conv_{1\times 1}(Cat(Conv_{3\times 3}(X)\;⊗A,Conv_{1\times 1}(X)⊗A,X)) \end{array}$$

**Figure 5:**
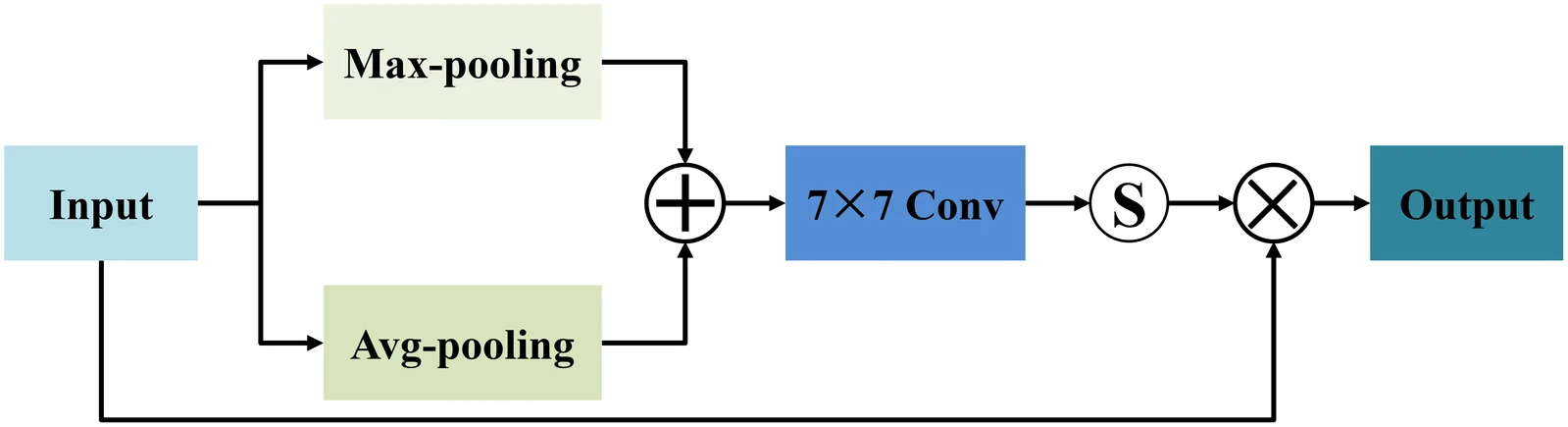
Structure of spatial attention module.

As shown in **Figure 5**, the spatial attention module (Cheng et al., 2022; Huang et al., 2022) is designed to emphasize informative spatial regions and suppress irrelevant background responses. Specifically, the input feature map is first processed by max-pooling and average-pooling operations in parallel to capture complementary spatial cues. The pooled features are then fused and passed through a 7×7 convolution followed by a Sigmoid activation to generate a spatial attention map. Finally, the obtained attention map is multiplied with the input feature map, enabling the network to focus more on lesion-related regions and enhance the representation of spatial details.

### 2.4 Cross-level feature alignment module

**Figure 6:**
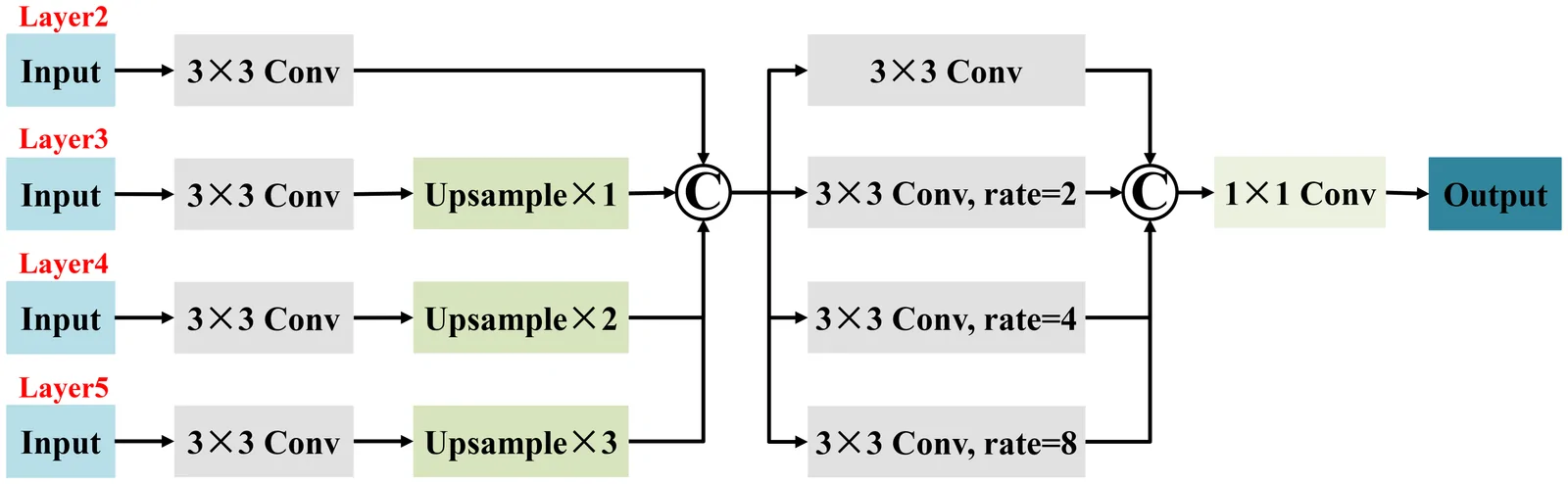
Structure of cross-level feature alignment module. Taking the Layer2 skip connection as an example, the encoded features from the Layer2, Layer3, Layer4, and Layer5 are aggregated and fused.

Although the skip connections in conventional U-Net can effectively transmit encoder features to the decoder, they usually adopt a one-to-one fusion manner, where only features from the corresponding encoder stage are directly concatenated with decoder features. Such a design ignores the complementary information among different encoder levels and cannot effectively bridge the semantic gap between shallow and deep representations. To address this issue, we introduce a cross-level feature alignment module into the second, third, and fourth skip connections of ACLA-Net, as shown in **Figure 1**. The main purpose of CLFAM is to aggregate and align encoder features from multiple semantic levels before they are transmitted to the decoder. By doing so, the decoder can benefit not only from the features of the current stage, but also from richer contextual information from deeper encoder stages. **Figure 6** illustrates the structure of CLFAM by taking the Layer2 skip connection as an example. Specifically, the encoded features from Layer2, Layer3, Layer4, and Layer5 are first processed by separate 3×3 convolutions to refine their feature representations. Since these features have different spatial resolutions, the features from Layer3, Layer4, and Layer5 are then upsampled to the same resolution as that of Layer2. After spatial alignment, all the features are concatenated along the channel dimension to form a unified multi-level representation. To further enhance the discriminative ability of the aggregated features, the concatenated feature map is passed through several parallel 3×3 convolutions with different dilation rates. In this way, CLFAM can capture contextual information under multiple receptive fields and better model lesion regions with varying sizes and shapes. The outputs of these convolutions are then concatenated again and fused by a 1×1 convolution to generate the final output feature of the skip connection. Unlike conventional one-to-one skip connections that rely only on features from the corresponding encoder stage, CLFAM aggregates and aligns features from multiple encoder levels and further refines them through multi-scale dilated convolutions. This design reduces the semantic discrepancy among hierarchical features and provides the decoder with richer and more discriminative contextual representations. The overall computation process of CLFAM is formulated in Equations (3) and (4):

(3) $$Y=Conv_{1\times 1}\;(Cat(Conv_{3\times 3}(F_{c}),DConv_{3\times 3}^{r=2}(F_{c}),DConv_{3\times 3}^{r=4}(F_{c}),DConv_{3\times 3}^{r=8}(F_{c})))$$

Where

(4) $$F_{c}\;=Cat(Conv_{3\times 3}(X_{2}),Up_{1}\;(Conv_{3\times 3}(X_{3})),Up_{2}\;(Conv_{3\times 3}(X_{4})),Up_{3}\;(Conv_{3\times 3}(X_{5})))$$

where $X_{2}$, $X_{3}$, $X_{4}$ and $X_{5}$ denote the encoder features from Layer2 to Layer5.

### 2.5 SE-guided context aggregation module

**Figure 7:**
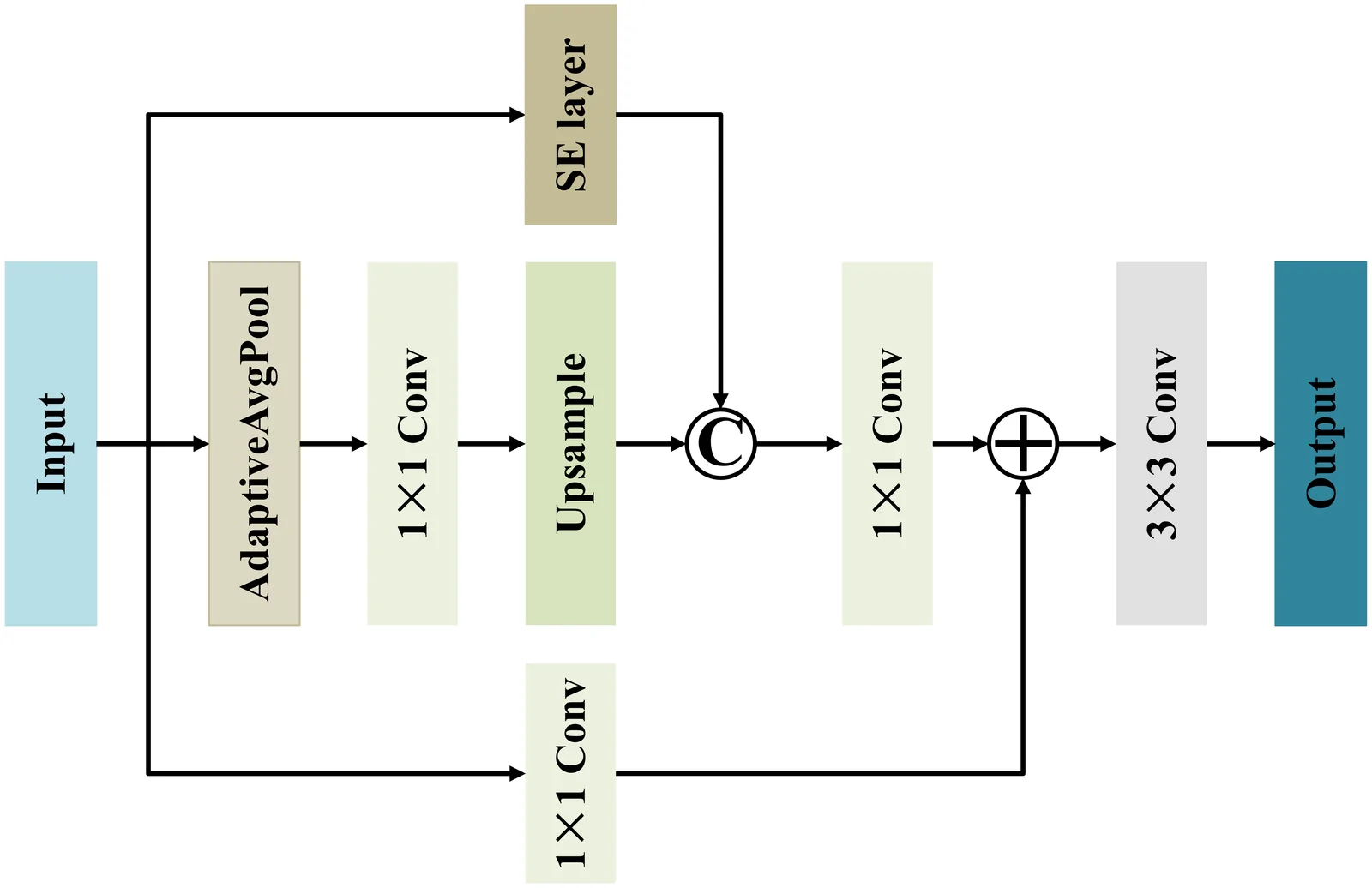
Structure of SE-guided context aggregation module.

At the bottleneck stage, the extracted features contain rich high-level semantic information, which is crucial for accurate prostate cancer lesion segmentation. However, due to the large variations in lesion morphology and the often ambiguous boundaries between tumors and surrounding tissues, conventional convolutional operations are still limited in capturing sufficient global contextual dependencies. To address this issue, a SE-guided context aggregation module is introduced at the bottleneck stage, as shown in **Figure 7**. Specifically, given an input feature map, one branch first applies adaptive average pooling followed by a 1×1 convolution and an upsampling operation to extract and restore global contextual information. Meanwhile, another branch employs an SE layer to adaptively recalibrate channel-wise responses and emphasize informative feature channels. The outputs of these two branches are then concatenated and fused by a 1×1 convolution, enabling the module to combine global context with channel-aware feature representations. In addition, a residual branch with a 1×1 convolution is introduced to preserve the original semantic information of the input feature and facilitate stable feature propagation. After that, the fused contextual feature is added to the residual feature, and a 3×3 convolution is further applied to refine the integrated representation and generate the final output. Unlike conventional bottleneck convolutions, SECAM combines global context aggregation with channel-wise feature recalibration and residual information preservation, thereby enhancing high-level semantic representation and providing richer contextual cues for the decoder. The overall process of SECAM is defined in Equation (5):

(5) $$Y=Conv_{3\times 3}(Conv_{1\times 1}(Cat(Up(Conv_{1\times 1}(AAP(X))),SE(X)))+Conv_{1\times 1}(X))$$

where AAP denotes adaptive average pooling.

### 2.6 Loss function

In this study, the Dice loss (Luo et al., 2024; Yuan et al., 2024) is adopted as the optimization objective for training ACLA-Net. Compared with conventional pixel-wise loss functions, Dice loss is more suitable for medical image segmentation tasks, especially when the target region occupies only a small proportion of the image. Since prostate cancer lesions are usually small and irregular in shape, there often exists a severe imbalance between lesion pixels and background pixels. The Dice loss is defined in Equation (6):

(6) $$L_{Dice}=1-\frac{2\sum_{i=1}^{N}y_{i}{\hat{y}}_{i}}{\sum_{i=1}^{N}y_{i}^{2}+\sum_{i=1}^{N}{\hat{y}}_{i}^{2}}$$

where $y_{i}$ and ${\hat{y}}_{i}$ denote the predicted value and ground-truth label of the *i*-th pixel, N is the total number of pixels.

## 3 Experiments and discussion

### 3.1 Dataset

**Figure 8:**
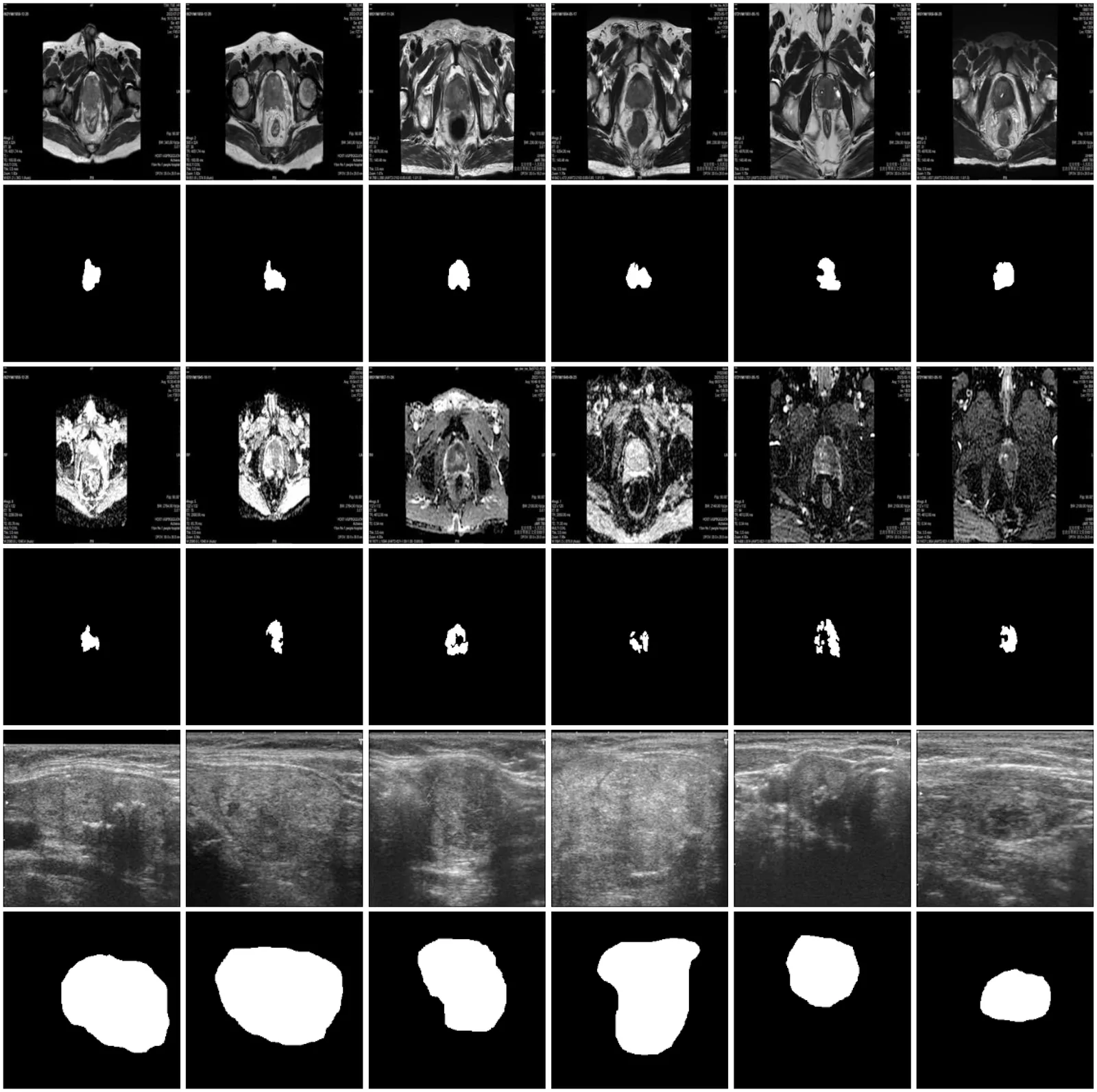
Representative examples from the ProstateCancer-T2WI, ProstateCancer-ADC, and DDTI datasets. The first two rows show the original images and corresponding annotations from ProstateCancer-T2WI, the third and fourth rows present those from ProstateCancer-ADC, and the last two rows show those from the DDTI dataset.

To evaluate the performance and generalization ability of the proposed ACLA-Net, experiments were conducted on two self-constructed prostate cancer MRI datasets and one publicly available thyroid ultrasound dataset. The two prostate cancer MRI datasets, namely ProstateCancer-T2WI and ProstateCancer-ADC, were retrospectively constructed from an overall cohort of 201 patients at Yibin First People’s Hospital. MRI examinations were performed using a 3.0-T MRI scanner (Philips, Amsterdam, the Netherlands) with a 32-channel phased-array body coil for signal reception, with the center of the coil positioned approximately 5 cm above the pubic symphysis. ProstateCancer-T2WI consists of T2-weighted imaging (T2WI) scans, whereas ProstateCancer-ADC consists of apparent diffusion coefficient (ADC) images. During dataset construction, images with poor image quality or insufficient lesion visibility were excluded to ensure reliable annotation and evaluation. Each lesion image was manually annotated by four experienced clinicians. The annotators collaboratively delineated the lesion regions at the pixel level, and any disagreements were resolved through discussion and consensus to obtain the final ground-truth masks for supervised learning and performance evaluation. To avoid patient-level data leakage and reduce the influence of highly correlated images from the same examination, all data were partitioned strictly at the patient level. Patient IDs were randomly assigned to the training, validation, and test subsets, after which all corresponding images from the same patient were assigned exclusively to the same subset. Thus, no patient appeared simultaneously in the training, validation, and test sets. Specifically, the ProstateCancer-T2WI dataset contains 1030 images, including 618 images for training, 206 images for validation, and 206 images for testing, whereas the ProstateCancer-ADC dataset contains 890 images, including 534 images for training, 178 images for validation, and 178 images for testing. In addition, the publicly available DDTI thyroid ultrasound dataset was introduced as an external validation dataset to further evaluate the generalization ability of ACLA-Net across different anatomical regions and imaging modalities. The DDTI dataset contains 637 thyroid ultrasound images acquired using a single ultrasound imaging device, with pixel-level segmentation masks provided for each image; among them, 383 images were used for training, 127 images for validation, and 127 images for testing. Representative examples from the ProstateCancer-T2WI, ProstateCancer-ADC, and DDTI datasets are shown in **Figure 8**.

### 3.2 Evaluation metrics

To quantitatively assess the segmentation performance of the proposed method, three widely used evaluation metrics were adopted in this study, including Dice (Wan et al., 2025a; Li et al., 2022), Matthews correlation coefficient (MCC) (Wan et al., 2025b; Shang et al., 2024), and Jaccard (Yang et al., 2024; Yeung et al., 2023). These metrics evaluate the similarity and consistency between the predicted segmentation results and the corresponding ground-truth annotations from different perspectives, thus providing a comprehensive assessment of model performance. The Dice, MCC, and Jaccard metrics are defined in Equations (7)–(9):

(7) $$Dice=\frac{2TP}{2TP+FN+FP}$$

(8) $$MCC=\frac{TP\times TN-FP\times FN}{\sqrt{\left(TP+FN)(TP+FP)(TN+FN)(TN+FP\right)}}$$

(9) $$Jaccard=\frac{TP}{TP+FN+FP}$$

### 3.3 Implementation details

The proposed ACLA-Net was developed using the PyTorch 2.0.0 framework. All experiments were carried out on a workstation equipped with an NVIDIA GeForce RTX A6000 GPU with 48 GB memory. For network optimization, the Adam optimizer was employed, with the initial learning rate to 1×10^−^³. The training process was conducted for 200 epochs using a batch size of 16. Prior to training, all images were resized to 256×256 pixels. Apart from image resizing, no additional preprocessing procedures were applied. No data augmentation techniques were applied during training, validation, or testing. During inference, the predicted probability maps were binarized using a threshold of 0.5; pixels with predicted values greater than 0.5 were assigned to the foreground, while the remaining pixels were assigned to the background. No additional post-processing operations were performed on the predicted segmentation masks. To ensure reproducibility, a fixed random seed was applied to PyTorch, CUDA, NumPy, and Python random operations, and `torch.backends.cudnn.deterministic` was set to `True` to enforce deterministic computation. These experimental settings were consistently applied throughout all comparisons and analyses.

**Figure 9:**
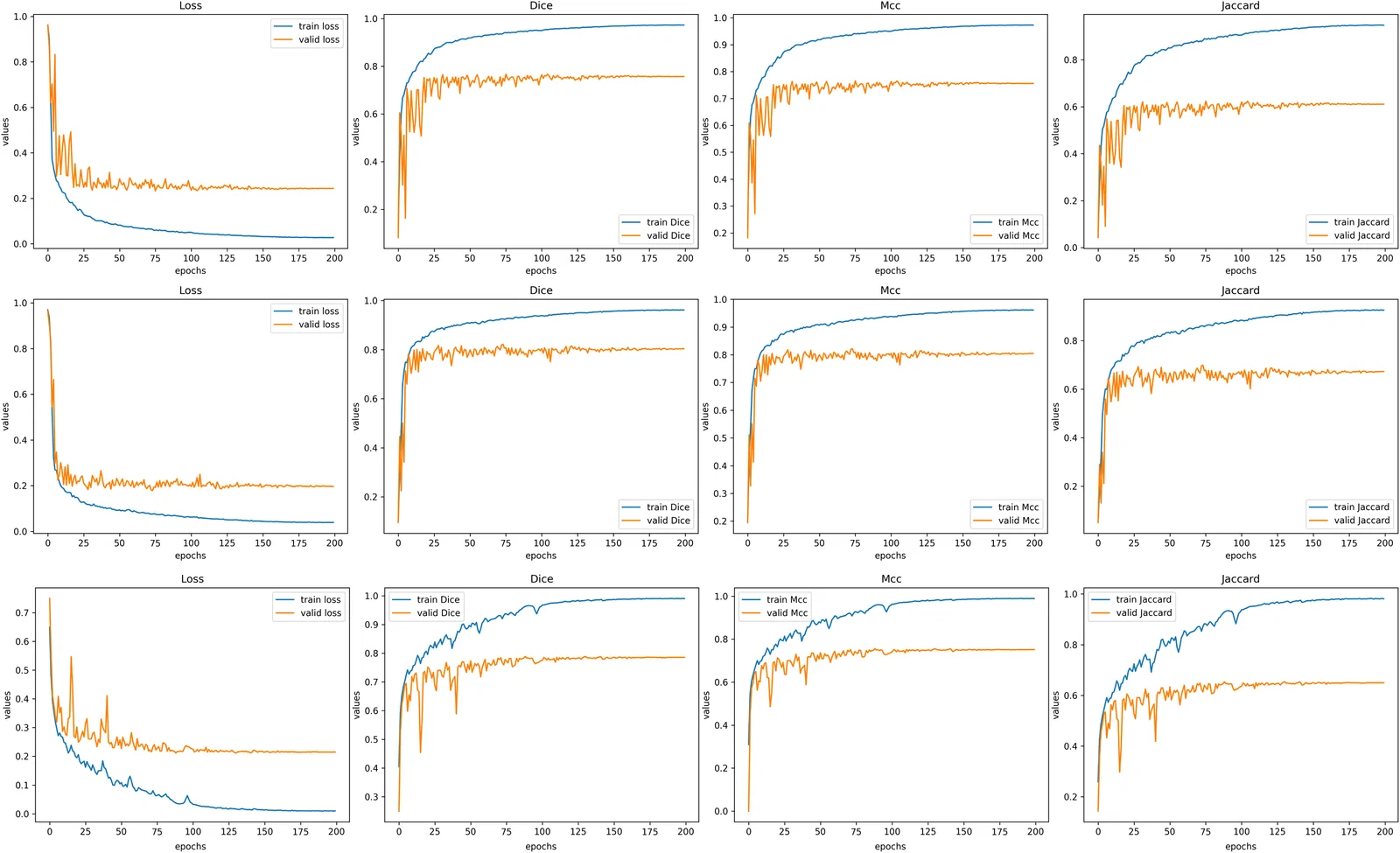
Training and validation curves of ACLA-Net on the ProstateCancer-T2WI, ProstateCancer-ADC, and DDTI datasets. The first row shows the curves of four evaluation metrics on ProstateCancer-T2WI, the second row presents the corresponding curves on ProstateCancer-ADC, and the third row shows those on the DDTI dataset.

**Figure 9** presents the training and validation curves of ACLA-Net on the ProstateCancer-T2WI, ProstateCancer-ADC, and DDTI datasets. As the number of training epochs increases, the training loss on all three datasets gradually decreases, while the Dice, MCC, and Jaccard scores continuously increase and eventually approach stable levels. The validation curves exhibit relatively noticeable fluctuations during the early training stage, particularly within the first several epochs, but gradually become stable as training progresses. Similar convergence trends can be observed across the three datasets, although a certain gap remains between the training and validation curves. Importantly, the validation metrics do not show a sustained decline in the later training stage, indicating that ACLA-Net achieves stable convergence and maintains relatively consistent segmentation performance on unseen validation data. Overall, these results demonstrate the stable optimization behavior of ACLA-Net across different datasets and imaging modalities.

### 3.4 Ablation study

**Figure 10:**
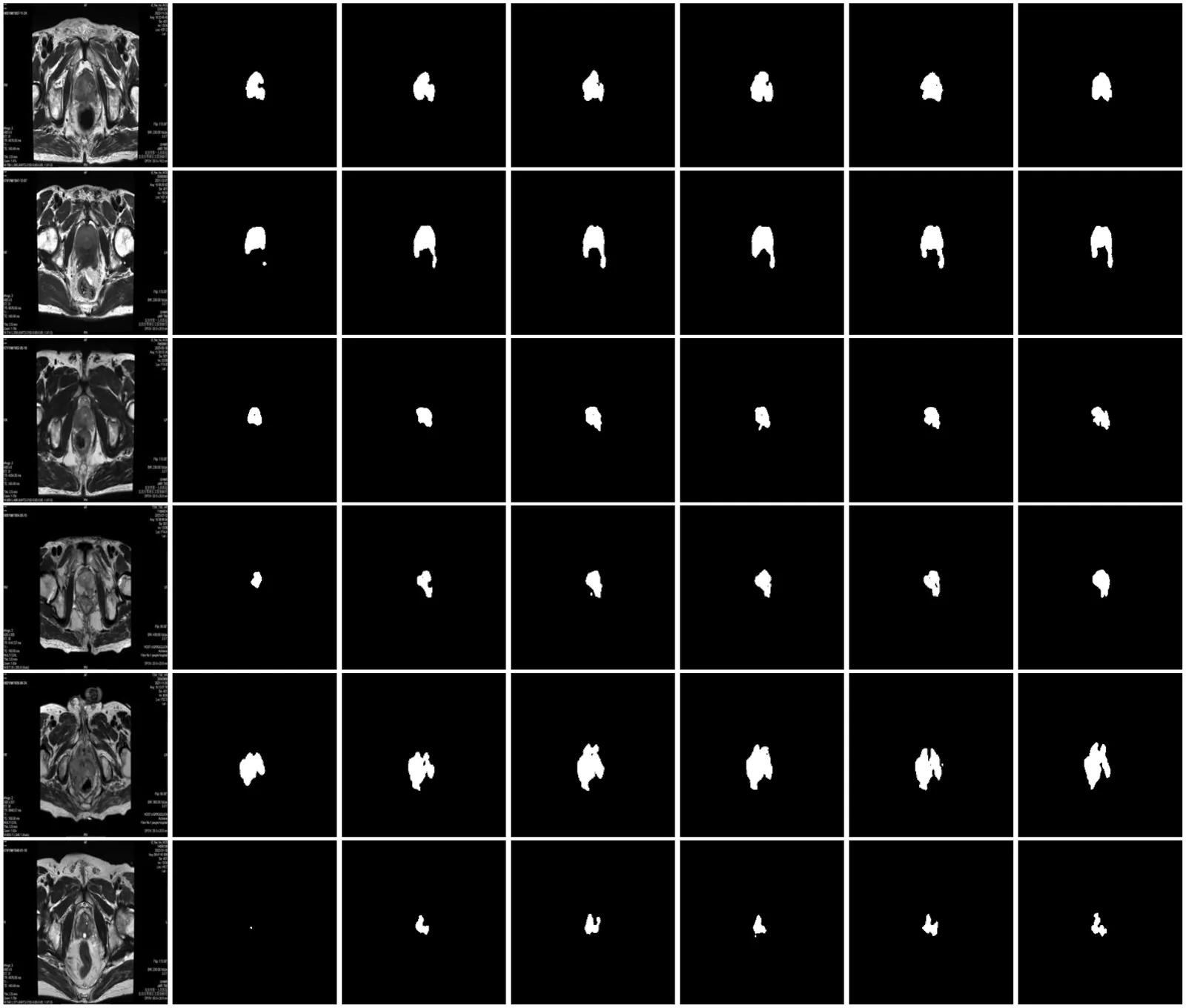
Visualization results of the ablation study on the ProstateCancer-T2WI dataset. The first two columns show the original images and corresponding annotations, while the remaining columns present the segmentation results of Baseline, Baseline+CLFAM, Baseline+CLFAM+SAFM, Baseline +CLFAM +SAFM+SECAM, and Baseline+CLFAM+SAFM+SECAM+SEMKDM.

To verify the effectiveness of each proposed component in ACLA-Net, an ablation study was conducted on the ProstateCancer-T2WI dataset, and the results are reported in **Table 1**. The baseline model was the conventional U-Net, and the proposed modules were progressively introduced, including CLFAM, SAFM, SECAM, and SEMKDM. As shown in **Table 1**, the baseline U-Net achieved Dice of 0.7006, MCC of 0.7117, and Jaccard of 0.5427. After introducing CLFAM, the performance improved noticeably to 0.7586 in Dice, 0.7580 in MCC, and 0.6128 in Jaccard. Compared with the baseline, the gains reached 0.0580, 0.0463, and 0.0701. These results indicate that aggregating and aligning encoder features across different levels can effectively enhance feature interaction and improve the utilization of complementary multi-scale information. When SAFM was further incorporated, the segmentation performance was further improved, with Dice, MCC, and Jaccard increasing to 0.7638, 0.7628, and 0.6204, respectively. This demonstrates that introducing spatial attention into the shallow skip connection helps the network emphasize informative spatial regions and preserve more useful boundary details. On this basis, adding SECAM further boosted the performance to 0.7669 in Dice, 0.7658 in MCC, and 0.6232 in Jaccard. Although the improvement is relatively moderate, the consistent gains suggest that SECAM can effectively enhance bottleneck features by aggregating global contextual information and strengthening high-level semantic representation. Finally, after replacing the conventional encoder blocks with SEMKDM, the complete ACLA-Net achieved the best performance, with Dice of 0.7798, MCC of 0.7782, and Jaccard of 0.6403. Compared with the previous configuration, SEMKDM brought additional improvements of 0.0129, 0.0124, and 0.0171, indicating that the proposed multi-kernel depthwise design is beneficial for extracting more discriminative multi-scale features. Overall, compared with the baseline U-Net, the final ACLA-Net improved Dice, MCC, and Jaccard by 0.0792, 0.0665, and 0.0976. These results confirm that each proposed module contributes positively to the final segmentation performance, and the combination of all modules yields the most effective network configuration. **Figure 10** further shows the visual results of the ablation study on the ProstateCancer-T2WI dataset. Compared with the baseline U-Net, the progressive introduction of CLFAM, SAFM, SECAM, and SEMKDM leads to more complete lesion regions, clearer boundaries, and better agreement with the ground truth. Among all configurations, the complete ACLA-Net produces the most accurate segmentation results, which is consistent with the quantitative improvements reported in **Table 1**.

**Table 1:** Ablation study results on the ProstateCancer-T2WI dataset.

Method	Dice	MCC	Jaccard
Baseline (U-Net)	0.7006 ± 0.0599	0.7117 ± 0.0547	0.5427 ± 0.0772
Baseline+CLFAM	0.7586 ± 0.0390	0.7580 ± 0.0393	0.6128 ± 0.0518
Baseline+CLFAM+SAFM	0.7638 ± 0.0489	0.7628 ± 0.0485	0.6204 ± 0.0635
Baseline+CLFAM+SAFM+SECAM	0.7669 ± 0.0346	0.7658 ± 0.0344	0.6232 ± 0.0458
Baseline+CLFAM+SAFM+SECAM+SEMKDM (ACLA-Net)	0.7798 ± 0.0328	0.7782 ± 0.0330	0.6403 ± 0.0450

### 3.5 Comparison study

**Table 2** presents a quantitative comparison between ACLA-Net and several representative segmentation methods on the ProstateCancer-T2WI dataset. The results are reported as mean ± standard deviation to provide a more comprehensive evaluation of both segmentation performance and stability. Among the compared methods, BMANet achieved the lowest performance, with Dice, MCC, and Jaccard scores of 0.6843, 0.6822, and 0.5215. W-Net and 2MSPK-Net obtained similar results, with Dice scores of 0.7306 and 0.7303, while DESENet, LMFR-Net, GAC-Net, BRRNet, and PMFSNet achieved Dice scores ranging from 0.7396 to 0.7452. Ultranet further improved the segmentation performance, achieving Dice, MCC, and Jaccard scores of 0.7645, 0.7629, and 0.6200. Among all baseline methods, PGA-Net achieved the best overall performance, with corresponding scores of 0.7693, 0.7685, and 0.6263. In contrast, the proposed ACLA-Net achieved the highest average performance across all three evaluation metrics, with Dice, MCC, and Jaccard scores of 0.7798, 0.7782, and 0.6403. Compared with the best-performing baseline PGA-Net, ACLA-Net yielded absolute improvements of 0.0105, 0.0097, and 0.0140 in Dice, MCC, and Jaccard. In addition, the standard deviations of ACLA-Net were 0.0328, 0.0330, and 0.0450 for the three metrics, which are relatively small and comparable to those of the strongest competing methods, indicating stable segmentation performance across repeated experiments. These results demonstrate that the proposed cross-level feature alignment and stage-specific feature enhancement strategy can effectively improve the segmentation of prostate cancer lesions on T2WI images.

**Table 2:** Comparative study on the ProstateCancer-T2WI dataset.

Method	Dice	MCC	Jaccard
W-Net (Tang et al., 2024)	0.7306 ± 0.0427	0.7305 ± 0.0425	0.5773 ± 0.0532
2MSPK-Net (Yue et al., 2025)	0.7303 ± 0.0422	0.7298 ± 0.0423	0.5768 ± 0.0518
DESENet (Tang et al., 2025)	0.7452 ± 0.0346	0.7437 ± 0.0353	0.5950 ± 0.0444
LMFR-Net (Zhang et al., 2025)	0.7437 ± 0.0422	0.7438 ± 0.0416	0.5938 ± 0.0535
PGA-Net (Dong et al., 2019)	0.7693 ± 0.0325	0.7685 ± 0.0329	0.6263 ± 0.0438
GAC-Net (Wu et al., 2025a)	0.7438 ± 0.0323	0.7438 ± 0.0310	0.5932 ± 0.0410
BRRNet (Shao et al., 2020)	0.7396 ± 0.0357	0.7387 ± 0.0355	0.5880 ± 0.0449
PMFSNet (Zhong et al., 2025)	0.7429 ± 0.0358	0.7418 ± 0.0356	0.5923 ± 0.0460
Ultranet (Han et al., 2025)	0.7645 ± 0.0340	0.7629 ± 0.0343	0.6200 ± 0.0450
BMANet (Wu et al., 2025b)	0.6843 ± 0.0401	0.6822 ± 0.0402	0.5215 ± 0.0463
ACLA-Net	0.7798 ± 0.0328	0.7782 ± 0.0330	0.6403 ± 0.0450

**Figure 11:**
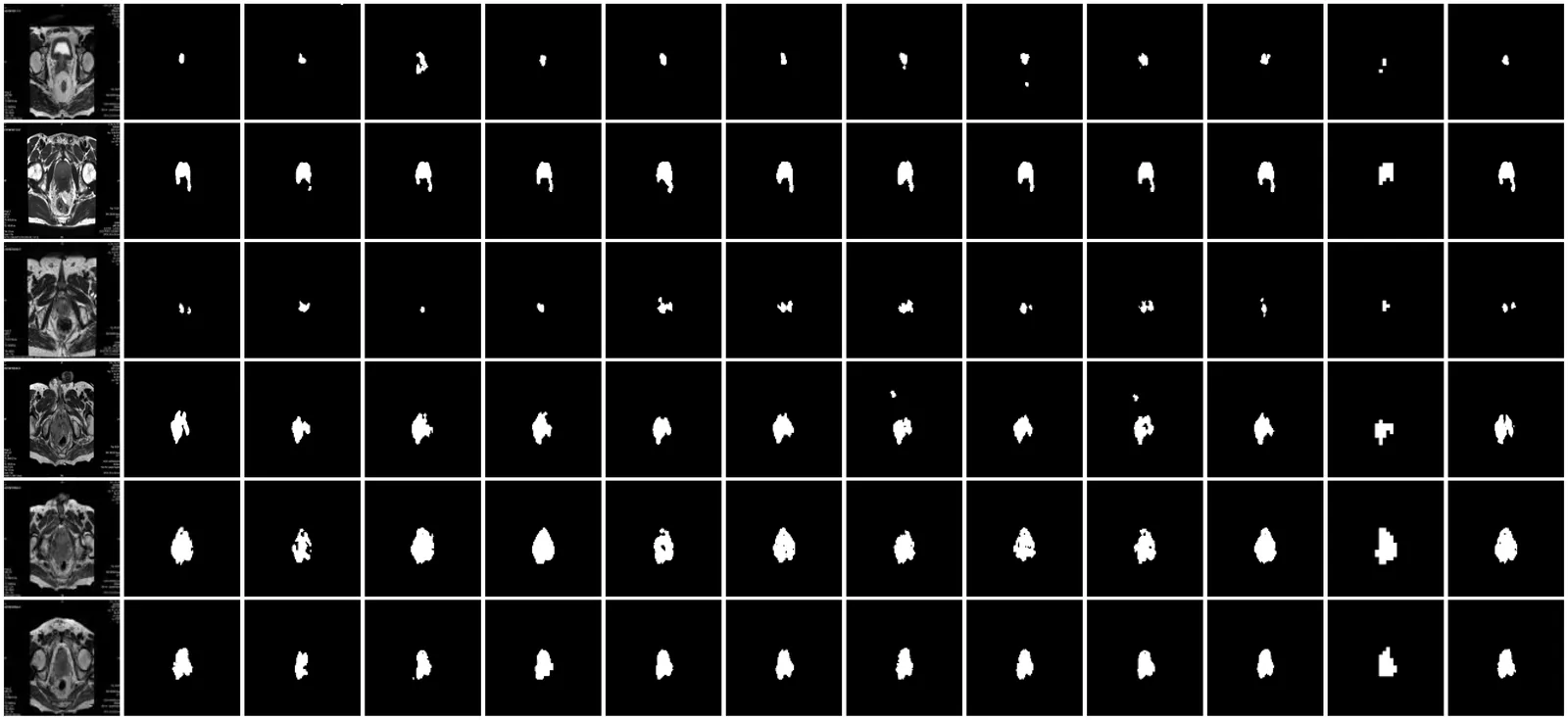
Visual comparison results of different methods on the ProstateCancer-T2WI dataset. The first two columns show the original images and corresponding annotations, while the remaining columns present the segmentation results of W-Net, 2MSPK-Net, DESENet, LMFR-Net, PGA-Net, GAC-Net, BRRNet, PMFSNet, Ultranet, BMANet, and ACLA-Net.

The visual comparison results on the ProstateCancer-T2WI dataset are presented in **Figure 11**. Overall, although all comparison methods can localize the lesion regions to some extent, clear differences can still be observed in terms of lesion completeness, boundary delineation, and shape consistency. W-Net and 2MSPK-Net are able to segment the main lesion regions, but they tend to produce incomplete predictions or inaccurate shapes in some challenging cases, especially for small lesions and irregular boundaries. DESENet and LMFR-Net show improved segmentation continuity by enhancing cross-resolution interaction and multi-scale feature fusion, respectively. However, their results still exhibit boundary deviation or insufficient sensitivity to subtle lesion structures. PGA-Net and GAC-Net benefit from pyramid/context modeling and attention mechanisms, and thus achieve relatively better visual performance, but they still suffer from slight over-segmentation or under-segmentation in difficult samples. BRRNet and PMFSNet further improve the lesion appearance through refinement and attention-based multi-scale enhancement, yet some predicted masks remain less compact or deviate from the ground truth in fine details. Ultranet generally provides relatively complete lesion predictions, but some boundary inaccuracies and shape deviations remain in challenging cases. In comparison, BMANet shows more noticeable under-segmentation or irregular predictions in several samples, particularly for small or morphologically complex lesions. In contrast, ACLA-Net generates segmentation results that are visually closer to the manual annotations in most cases. It not only preserves more complete lesion regions, but also produces clearer boundaries and better shape consistency, particularly for small targets and lesions with complex morphology. These advantages can be attributed to the joint effect of CLFAM, SAFM, SECAM, and SEMKDM, which enable more effective cross-level feature alignment, shallow detail preservation, contextual aggregation, and multi-scale feature extraction.

The quantitative comparison results on the ProstateCancer-ADC dataset are summarized in **Table 3**. Among the compared methods, BMANet achieves the lowest performance, with Dice, MCC, and Jaccard scores of 0.6927, 0.6919, and 0.5318. PMFSNet, PGA-Net, DESENet, and 2MSPK-Net obtain relatively lower Dice scores ranging from 0.7909 to 0.8044, while W-Net achieves a Dice score of 0.8099. LMFR-Net further improves the segmentation performance, achieving Dice, MCC, and Jaccard scores of 0.8203, 0.8201, and 0.6981. GAC-Net and BRRNet also demonstrate competitive performance, with Dice scores of 0.8259 and 0.8255. Among all baseline methods, Ultranet achieves the best overall performance, with Dice, MCC, and Jaccard scores of 0.8268, 0.8264, and 0.7070. In comparison, the proposed ACLA-Net achieves the highest average values across all three evaluation metrics, reaching 0.8332, 0.8328, and 0.7159 for Dice, MCC, and Jaccard. Compared with Ultranet, ACLA-Net yields absolute improvements of 0.0064, 0.0064, and 0.0089 in Dice, MCC, and Jaccard. Moreover, ACLA-Net obtains standard deviations of 0.0389, 0.0390, and 0.0562, which are lower than those of Ultranet (0.0437, 0.0437, and 0.0628), indicating more stable segmentation performance compared with the strongest baseline. These results demonstrate that ACLA-Net achieves a favorable balance between segmentation accuracy and stability on the ProstateCancer-ADC dataset, further confirming the effectiveness of the proposed network across different prostate cancer MRI modalities.

**Table 3:** Comparative study on the ProstateCancer-ADC dataset.

Method	Dice	MCC	Jaccard
W-Net (Tang et al., 2024)	0.8099 ± 0.0431	0.8091 ± 0.0426	0.6827 ± 0.0609
2MSPK-Net (Yue et al., 2025)	0.8044 ± 0.0577	0.8042 ± 0.0575	0.6767 ± 0.0827
DESENet (Tang et al., 2025)	0.8031 ± 0.0426	0.8021 ± 0.0429	0.6731 ± 0.0588
LMFR-Net (Zhang et al., 2025)	0.8203 ± 0.0468	0.8201 ± 0.0458	0.6981 ± 0.0458
PGA-Net (Dong et al., 2019)	0.8008 ± 0.0387	0.7999 ± 0.0387	0.6695 ± 0.0537
GAC-Net (Wu et al., 2025a)	0.8259 ± 0.0421	0.8251 ± 0.0422	0.7056 ± 0.0619
BRRNet (Shao et al., 2020)	0.8255 ± 0.0619	0.8257 ± 0.0499	0.7061 ± 0.0499
PMFSNet (Zhong et al., 2025)	0.7909 ± 0.0455	0.7903 ± 0.0450	0.6565 ± 0.0625
Ultranet (Han et al., 2025)	0.8268 ± 0.0437	0.8264 ± 0.0437	0.7070 ± 0.0628
BMANet (Wu et al., 2025b)	0.6927 ± 0.0465	0.6919 ± 0.0459	0.5318 ± 0.0531
ACLA-Net	0.8332 ± 0.0389	0.8328 ± 0.0390	0.7159 ± 0.0562

**Figure 12:**
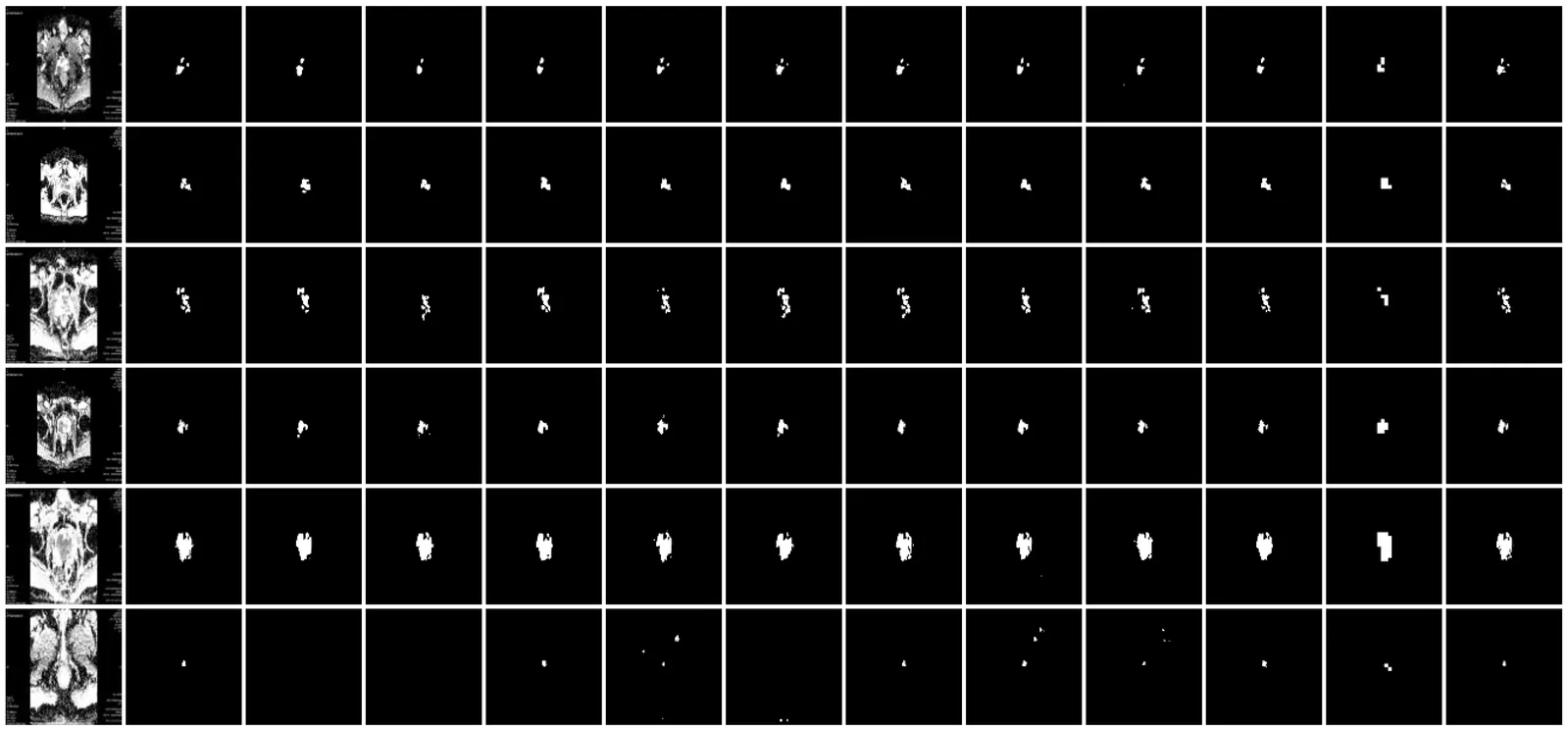
Visual comparison results of different methods on the ProstateCancer-ADC dataset. The first two columns show the original images and corresponding annotations, while the remaining columns present the segmentation results of W-Net, 2MSPK-Net, DESENet, LMFR-Net, PGA-Net, GAC-Net, BRRNet, PMFSNet, Ultranet, BMANet, and ACLA-Net.

**Figure 12** presents the visual comparison results on the ProstateCancer-ADC dataset. Overall, most competing methods can identify the main lesion regions, but noticeable differences remain in lesion completeness, boundary delineation, and suppression of false-positive regions. W-Net, 2MSPK-Net, DESENet, LMFR-Net, PGA-Net, GAC-Net, BRRNet, and PMFSNet generally capture the lesion locations, although some predictions exhibit incomplete contours, irregular shapes, or scattered false-positive responses in challenging cases. Ultranet produces relatively accurate and compact segmentation masks and shows better visual consistency than most baseline methods, whereas BMANet presents more obvious shape deviations and under-segmentation in several samples. In contrast, ACLA-Net generates predictions that are generally closer to the manual annotations, with more complete lesion regions, clearer boundaries, and fewer irrelevant responses. These qualitative observations are consistent with the quantitative results reported in **Table 3**, further demonstrating the effectiveness of ACLA-Net for prostate cancer lesion on ADC images.

The quantitative comparison results on the DDTI dataset are summarized in **Table 4**. Among the compared methods, Ultranet and LMFR-Net obtain relatively lower performance, with Dice scores of 0.6236 and 0.6343. 2MSPK-Net and BRRNet achieve similar results, with Dice scores of 0.6675 and 0.6684, while PMFSNet and GAC-Net further improve the Dice scores to 0.6859 and 0.6920. BMANet achieves Dice, MCC, and Jaccard scores of 0.7197, 0.6748, and 0.5641, whereas DESENet and PGA-Net obtain relatively competitive performance, reaching Dice scores of 0.7358 and 0.7407. Among all baseline methods, W-Net achieves the best overall performance, with Dice, MCC, and Jaccard scores of 0.7541, 0.7150, and 0.6081. In comparison, the proposed ACLA-Net achieves the highest average values across all three evaluation metrics, reaching 0.7752, 0.7438, and 0.6338 for Dice, MCC, and Jaccard. Compared with W-Net, ACLA-Net yields absolute improvements of 0.0211, 0.0288, and 0.0257 in Dice, MCC, and Jaccard. Moreover, ACLA-Net achieves standard deviations of 0.0270, 0.0229, and 0.0229, which are notably lower than those of W-Net (0.0527, 0.0519, and 0.0662), indicating more stable segmentation performance. These results demonstrate that ACLA-Net maintains favorable segmentation accuracy and stability on the DDTI dataset, supporting its generalization capability across different anatomical regions and imaging modalities.

**Table 4:** Comparative study on the DDTI dataset.

Method	Dice	MCC	Jaccard
W-Net (Tang et al., 2024)	0.7541 ± 0.0527	0.7150 ± 0.0519	0.6081 ± 0.0662
2MSPK-Net (Yue et al., 2025)	0.6675 ± 0.0593	0.6130 ± 0.0545	0.5038 ± 0.0648
DESENet (Tang et al., 2025)	0.7358 ± 0.0473	0.6942 ± 0.0481	0.5842 ± 0.0585
LMFR-Net (Zhang et al., 2025)	0.6343 ± 0.0828	0.5742 ± 0.0785	0.4696 ± 0.0851
PGA-Net (Dong et al., 2019)	0.7407 ± 0.0578	0.7047 ± 0.0579	0.5915 ± 0.0730
GAC-Net (Wu et al., 2025a)	0.6920 ± 0.0413	0.6415 ± 0.0368	0.5306 ± 0.0474
BRRNet (Shao et al., 2020)	0.6684 ± 0.0670	0.6196 ± 0.0691	0.5056 ± 0.0738
PMFSNet (Zhong et al., 2025)	0.6859 ± 0.0662	0.6351 ± 0.0664	0.5258 ± 0.0760
Ultranet (Han et al., 2025)	0.6236 ± 0.0506	0.5623 ± 0.0481	0.4551 ± 0.0537
BMANet (Wu et al., 2025b)	0.7197 ± 0.0449	0.6748 ± 0.0415	0.5641 ± 0.0535
ACLA-Net	0.7752 ± 0.0270	0.7438 ± 0.0229	0.6338 ± 0.0229

**Figure 13:**
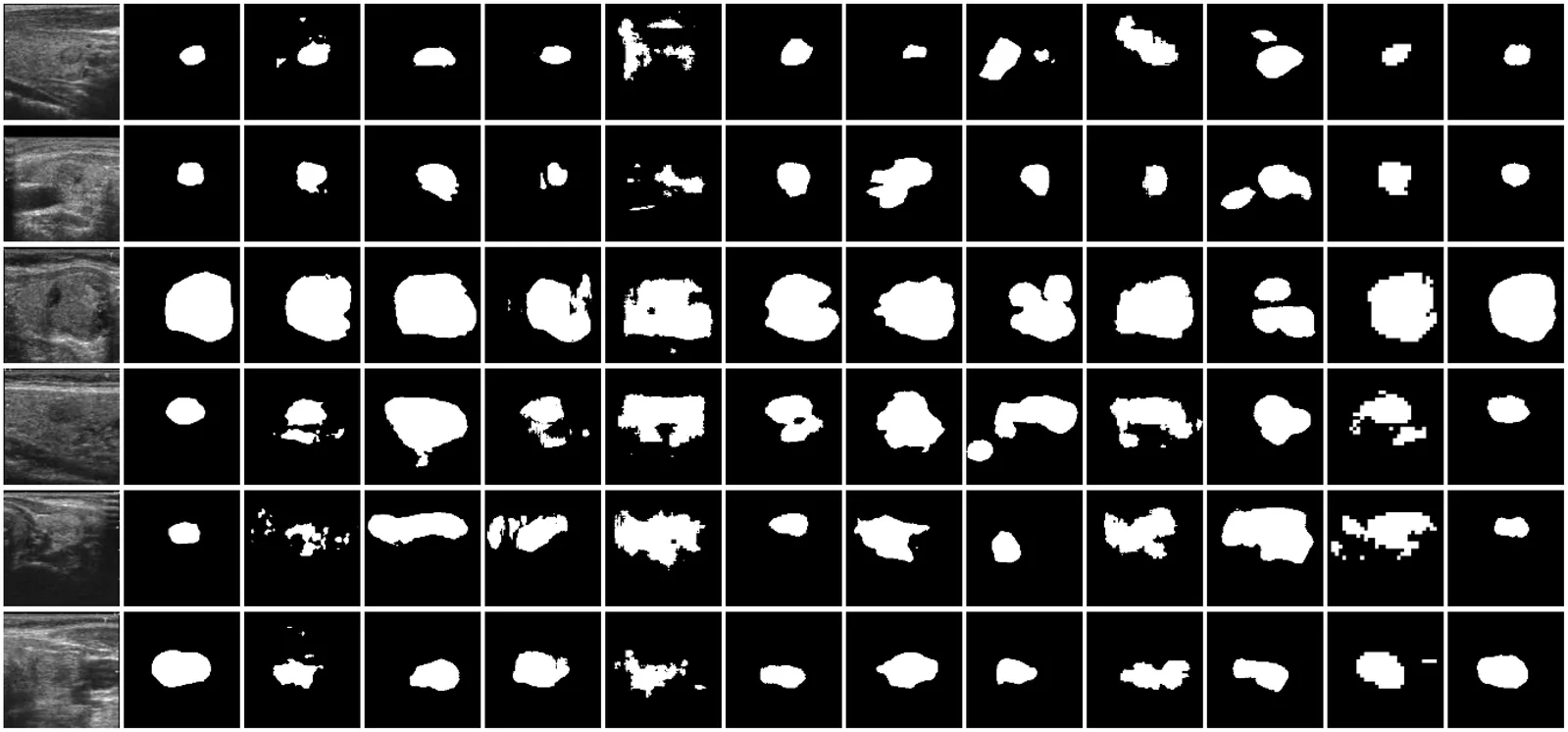
Visual comparison results of different methods on the DDTI dataset. The first two columns show the original images and corresponding annotations, while the remaining columns present the segmentation results of W-Net, 2MSPK-Net, DESENet, LMFR-Net, PGA-Net, GAC-Net, BRRNet, PMFSNet, Ultranet, BMANet, and ACLA-Net.

**Figure 13** presents the qualitative comparison results on the DDTI dataset. Compared with prostate MRI, thyroid ultrasound images exhibit stronger speckle noise, heterogeneous echogenicity, and less distinct lesion boundaries, which make accurate contour delineation more challenging. As shown in the figure, several competing methods can roughly identify the thyroid lesion regions, but their predictions are more easily affected by surrounding tissues and acoustic noise. In particular, some methods produce fragmented regions, irregular contours, or obvious over-segmentation when the lesion boundary is weak, while others tend to miss peripheral areas and generate incomplete masks. Although W-Net, DESENet, PGA-Net, and BMANet generally achieve more reasonable lesion localization, discrepancies in boundary shape and lesion extent can still be observed in several samples. In contrast, ACLA-Net shows better adaptability to the heterogeneous appearance of thyroid ultrasound images, producing more coherent lesion regions and predictions that more closely follow the manual annotations. The favorable performance on DDTI indicates that the proposed cross-level interaction and feature refinement mechanisms are not limited to prostate MRI, but can also effectively capture discriminative information under substantially different imaging characteristics, further supporting the generalization potential of ACLA-Net.

### 3.6 Analysis of computational efficiency

To further investigate the practical applicability of the proposed network, we compared the computational efficiency of ACLA-Net with several representative segmentation methods on the ProstateCancer-T2WI dataset, as summarized in **Table 5**. Four indicators, namely GFLOPs, Params, FPS, and inference time per image, were adopted to evaluate the computational complexity, parameter scale, and inference efficiency of different models. Among the compared methods, Ultranet is the most lightweight model, requiring only 0.56 GFLOPs and 0.17 M parameters, while achieving 55.27 FPS with an inference time of 18.09 ms per image. PMFSNet also exhibits low computational complexity, with 0.59 GFLOPs, 0.33 M parameters, and an inference time of 7.85 ms per image. LMFR-Net has the smallest parameter scale of only 0.07 M and achieves 135.11 FPS, corresponding to 7.40 ms per image. In contrast, 2MSPK-Net, PGA-Net, GAC-Net, and BRRNet involve considerably higher computational costs, with GFLOPs ranging from 58.79 to 81.36. Although BRRNet achieves the highest inference speed of 159.30 FPS, its computational cost remains relatively high at 58.79 GFLOPs. BMANet requires 9.19 GFLOPs and 28.40 M parameters, while achieving 37.20 FPS with an inference time of 26.88 ms per image. For the proposed ACLA-Net, the computational cost is 6.83 GFLOPs with 3.66 M parameters, while the inference speed reaches 93.79 FPS, corresponding to 10.66 ms per image. Overall, ACLA-Net maintains relatively low computational complexity and parameter overhead while providing satisfactory inference efficiency, demonstrating a favorable balance between model complexity and running speed.

**Table 5:** Computational efficiency on the ProstateCancer-T2WI dataset.

Method	GFLOPs	Params(M)	FPS	Inference time (ms/image)
W-Net (Tang et al., 2024)	11.91	29.00	5.96	167.79
2MSPK-Net (Yue et al., 2025)	59.95	28.36	47.91	20.87
DESENet (Tang et al., 2025)	3.17	1.16	46.02	21.73
LMFR-Net (Zhang et al., 2025)	3.49	0.07	135.11	7.40
PGA-Net (Dong et al., 2019)	60.25	34.35	136.33	7.34
GAC-Net (Wu et al., 2025a)	81.36	31.89	7.49	133.51
BRRNet (Shao et al., 2020)	58.79	14.93	159.30	6.26
PMFSNet (Zhong et al., 2025)	0.59	0.33	127.35	7.85
Ultranet (Han et al., 2025)	0.56	0.17	55.27	18.09
BMANet (Wu et al., 2025b)	9.19	28.40	37.20	26.88
ACLA-Net	6.83	3.66	93.79	10.66

### 3.7 Optimizer selection

To investigate the influence of different optimization strategies on segmentation performance, six optimizers were evaluated on the ProstateCancer-T2WI dataset, including Adam, Adamax, NAdam, RMSprop, Rprop, and SGD. The corresponding results are listed in **Table 6**. As shown in **Table 6**, Adam achieves the best overall performance, with Dice of 0.7798, MCC of 0.7782, and Jaccard of 0.6403. RMSprop ranks second, obtaining 0.7755 in Dice, 0.7739 in MCC, and 0.6347 in Jaccard. NAdam and Rprop show similar performance, while Adamax and SGD yield relatively lower results. In particular, SGD achieves the lowest performance among all compared optimizers, with Dice of 0.7453, MCC of 0.7434, and Jaccard of 0.5950. Overall, the results indicate that Adam is the most suitable optimizer for the proposed ACLA-Net on the ProstateCancer-T2WI dataset. Its superior performance suggests that it can provide more effective parameter updates and better optimization stability, thereby leading to more accurate prostate cancer lesion segmentation.

**Table 6:** Optimizer selection on the ProstateCancer-T2WI dataset.

Optimizer	Dice	MCC	Jaccard
Adam	0.7798 ± 0.0328	0.7782 ± 0.0330	0.6403 ± 0.0450
Adamax	0.7497 ± 0.0377	0.7479 ± 0.0380	0.6011 ± 0.0489
NAdam	0.7712 ± 0.0372	0.7698 ± 0.0372	0.6291 ± 0.0504
RMSprop	0.7755 ± 0.0348	0.7739 ± 0.0353	0.6347 ± 0.0473
Rprop	0.7711 ± 0.0361	0.7693 ± 0.0365	0.6288 ± 0.0479
SGD	0.7453 ± 0.0318	0.7434 ± 0.0318	0.5950 ± 0.0404

## 4 Conclusion

In this paper, we proposed ACLA-Net, an attention-guided cross-level alignment network for MRI-based prostate cancer lesion segmentation. Built upon the U-Net architecture, the proposed network was designed to improve segmentation performance by enhancing cross-level feature interaction, refining shallow spatial information, and strengthening high-level contextual representation. Specifically, CLFAM was introduced into the second, third, and fourth skip connections to promote the alignment and fusion of encoder features across different semantic levels, while SAFM was incorporated into the first skip connection to emphasize discriminative spatial cues and improve shallow feature fusion. In addition, SECAM was employed at the bottleneck stage to aggregate richer global contextual information, and the conventional encoder blocks were replaced by SEMKDM to enhance multi-scale feature extraction with reduced computational redundancy. Extensive experiments were conducted on two self-constructed prostate cancer MRI datasets, as well as the public DDTI thyroid ultrasound dataset. The proposed ACLA-Net achieved Dice, MCC, and Jaccard scores of 0.7798, 0.7782, and 0.6403 on ProstateCancer-T2WI, and 0.8332, 0.8328, and 0.7159 on ProstateCancer-ADC. The experimental results demonstrate the effectiveness of ACLA-Net for prostate cancer lesion segmentation and provide preliminary evidence of cross-dataset and cross-modality generalization on the external DDTI dataset. However, several limitations remain. The prostate cancer MRI datasets used in this study were collected from a single medical center, with relatively consistent scanner settings and acquisition protocols, which may limit the diversity of imaging characteristics and patient populations represented in the current evaluation. Therefore, the performance of ACLA-Net on data acquired from other institutions, scanners, and heterogeneous imaging protocols has not yet been fully established. Although the DDTI dataset was introduced for external validation, it differs from prostate MRI in both anatomical region and imaging modality and therefore cannot substitute for direct external validation on independent prostate MRI cohorts. Further evaluation on independent multi-center prostate MRI datasets acquired using different scanners and imaging protocols is still necessary. In addition, the current framework is based on two-dimensional image segmentation and does not fully exploit the spatial continuity among adjacent MRI slices. In future work, we will further validate ACLA-Net on larger multi-center datasets and explore multimodal and three-dimensional prostate MRI analysis to improve its robustness and clinical applicability.

## Data Availability

The raw data supporting the conclusions of this article will be made available by the authors, without undue reservation.
